# Impact of cardiovascular-kidney-metabolic syndrome staging on clinical outcomes and management of acute pulmonary embolism: A comprehensive analysis

**DOI:** 10.1016/j.ijcha.2025.101831

**Published:** 2025-10-30

**Authors:** Shay Zvi Cherevatsky, Marlon V. Gatuz, Adam Folman, Maguli S. Barel, Rami Abu-Fanne, Dmitry Abramov, Mamas A. Mamas, Ariel Roguin, Ofer Kobo

**Affiliations:** a Rappaport Faculty of Medicine, Technion Israel Institute of Technology, Haifa, Israel; b Department of Cardiology, Hillel Yaffe Medical Center, Hadera, Israel; c Department of Cardiology, Linda Loma University Health, Linda Loma, USA; d Keele Cardiovascular Research Group, Keele University, UK; e National Institute for Health and Care Research (NIHR) Birmingham Biomedical Research Centre, UK

**Keywords:** Pulmonary embolism, Cardiovascular-kidney-metabolic syndrome, Risk factors, Outcomes

## Abstract

**Background:**

Pulmonary embolism (PE) is a life-threatening condition with high morbidity and mortality rates. Cardiovascular-Kidney-Metabolic (CKM) syndrome, representing a complex interplay of cardiovascular disease, kidney dysfunction, and metabolic disorders, may significantly impact PE outcomes. This study investigates the influence of CKM syndrome staging on clinical outcomes and management strategies in acute PE patients.

**Methods:**

This retrospective study analyzed 725,725 adult patients hospitalized with a primary diagnosis of PE between 2016 and 2019 using the National Inpatient Sample database. Patients were categorized into five CKM groups (0,1,2/3,4a, 4b) based on staging criteria. Multivariable logistic regression models were used to assess the relationship between in-hospital outcomes and CKM stages.

**Results:**

As CKM stages advanced, patients exhibited distinct profiles characterized by older age, male predominance and a higher prevalence of comorbidities. Multivariate analysis revealed that advanced CKM stages were less likely to receive invasive treatments (systemic thrombolysis: aOR 0.86, 95 % CI 0.81–0.92, p < 0.001) but had higher odds of adverse outcomes, including MACCE (aOR 1.53, 95 % CI 1.45–1.60, p < 0.001), mortality (aOR 1.33, 95 % CI 1.25–1.41, p < 0.001), and major bleeding (aOR 1.15, 95 % CI 1.08–1.23, p < 0.001). All odds ratios were computed using CKM stage 0 as the reference group.

**Conclusion:**

CKM syndrome staging significantly impacts clinical outcomes and management strategies in patients with PE. Advanced CKM stages are associated with higher risks of adverse events, including increased mortality and major bleeding complications. Paradoxically, these high-risk patients were less likely to receive invasive treatments, highlighting a critical gap in care.

## Highlights

• Advanced CKM stages worsen outcomes in pulmonary embolism patients.
• High CKM stages linked to less use of invasive PE treatments.
• CKM staging predicts PE mortality, bleeding, and adverse events.
• PE patients with CKM have longer hospital stays and higher costs.
• Study of 725,000 PE patients reveals care gaps by CKM stage.

## 1 Introduction

Pulmonary embolism (PE) is a life-threatening condition, with an estimated annual incidence of 60–120 cases per 100,000 individuals. [1,2]. The risk of PE increases markedly with age, particularly after 55 years [3]. Multiple factors predispose individuals to PE development, including prolonged immobility, active malignancy, pregnancy, oral contraceptive use, hormone replacement therapy, smoking inherited thrombophilias, but also concurrent cardiovascular comorbidities including obesity, diabetes, and chronic kidney disease (CKD), and chronic medical conditions such as heart failure [1,[4], [5], [6]].

Cardiovascular-Kidney-Metabolic (CKM) syndrome represents a complex interplay of cardiovascular disease, kidney dysfunction, and metabolic disorders such as diabetes and obesity [7] and is common in community cohorts [8]. Recent guidelines by the American Heart Association [7] define CKM syndrome as an integrative construct composed of interrelated cardiovascular, kidney, and metabolic disorders, reflecting real-world multimorbidity encountered in practice. Although the CKM staging system is still emerging and not yet incorporated into routine clinical workflow universally, it aims to provide a pragmatic risk stratification tool complementary to established single-disease risk scores by emphasizing cumulative organ system impairments and shared pathophysiology [7]. We acknowledge that familiarity with CKM staging is currently less widespread than with traditional indices; therefore, our analysis includes both a description of its components and an evaluation of its incremental prognostic value in acute pulmonary embolism management.

The Contributors to the CKM syndrome synergistically elevate the risk of adverse outcomes, encompassing cardiovascular events, renal dysfunction, and other complications [9. The American Heart Association introduced a novel staging construct to improve the multidisciplinary approach to prevention, risk stratification and management of these interconnected disorders. The staging system categorizes CKM syndrome progression from Stage 0 (no risk factors) to Stage 4 (established cardiovascular disease with or without kidney failure) [7].

CKM syndrome and PE are closely linked through shared risk factors. Individuals with CKM syndrome often experience elevated blood pressure, obesity, diabetes, and kidney dysfunction, all of which increase the likelihood of developing deep vein thrombosis (DVT), the primary cause of PE [10]. The presence of metabolic disorders and cardiovascular disease further exacerbates the risk of clot formation due to factors like increased blood viscosity, endothelial dysfunction, and reduced mobility associated with obesity or heart failure​. Moreover, kidney disease can impair clotting regulation and contribute to conditions like hypercoagulability, further elevating PE risk​.

There are limited prior data on the association of comorbidity profiles with the management and outcomes of patients with PE, particularly based on CKM staging. Therefore, we sought to investigate the impact of CKM syndrome stages on PE outcomes among those hospitalized for PE.

## 2 Methods

### 2.1 Data source

The National Inpatient Sample (NIS), available since 1988, is one of the most widely recognized publicly accessible inpatient healthcare databases in the United States. It collects data on approximately 7 million hospital stays each year through a 20 % stratified sample of discharges from U.S. community hospitals, excluding rehabilitation and long-term acute care facilities. As part of the Healthcare Cost and Utilization Project (HCUP), the NIS is designed to provide estimates of healthcare utilization and outcomes at both regional and national levels across the United States [11].

### 2.2 Study design and population

This retrospective study analyzed adult patients (aged 18 years and older) hospitalized with a primary diagnosis of PE between 2016 and 2019. Patients were categorized based on their CKM syndrome stages. Selection was based on diagnosis codes from the International Classification of Diseases, Tenth Revision, Clinical Modification (ICD-10-CM), implemented in 2016, which provided more detailed information compared to the previous ICD-9 system. Table S1 lists the ICD-10 codes used to define patient and procedural characteristics.

**Fig. 1:**
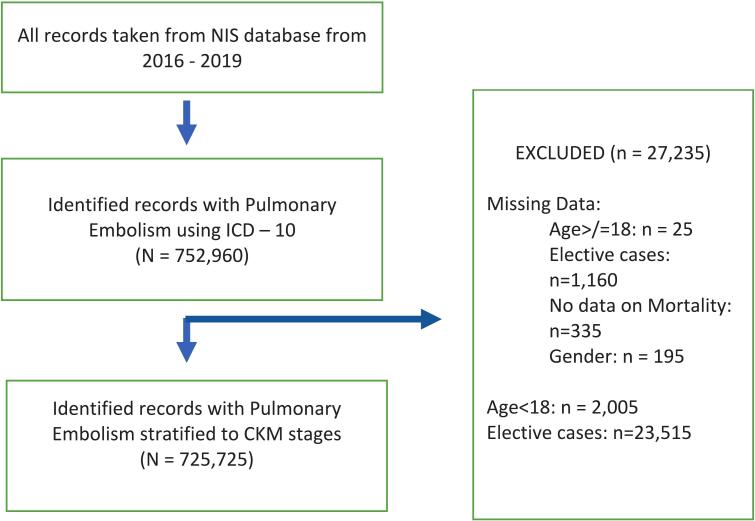
Flow diagram.

Demographic information, including age group, gender, ethnicity, day of admission (weekday or weekend), expected payer, and median household income by ZIP code, was recorded for each hospital discharge. Cases with missing data on age, gender, elective status, admission type and day, or mortality status were excluded from the analysis (see Fig. 1 for the study flow diagram). Each discharge record contained data on up to 30 diagnoses.

CKM syndrome, defined as the intricate relationship between cardiovascular disease, kidney disease, obesity, and type 2 diabetes, has been categorized by the American Heart Association (AHA) using a staging system ranging from Stage 0 to Stage 4 [7]. CKM stage assignment also used ICD-10 codes to identify: [1] cardiovascular diagnoses (e.g., coronary artery disease, heart failure), [2] kidney dysfunction (e.g., CKD, ESRD), and [3] metabolic risk factors (e.g., type 2 diabetes, obesity). Stage definitions are detailed in Supplementary Table S2. In this framework Stage 0 represents individuals with no CKM risk factors, while Stage 1 includes early risk factors such as abdominal obesity and prediabetes. We combined Stages 2 and 3 due to the difficulty in distinguishing asymptomatic CVD in Stage 3. This combined category covers both metabolic conditions like type 2 diabetes and chronic kidney disease, as well as potential subclinical cardiovascular or kidney disease. Stage 4 is further divided into 4a (established cardiovascular disease without kidney failure) and 4b (with kidney failure). Furthermore, in this study, a “high-risk PE” was defined as PE with cardiogenic shock, mechanical ventilation, mechanical circulatory support (MCS), or vasopressors [12,13]. A comprehensive list of ICD-10-CM codes used is provided in the Supplementary Table S1. These codes were also applied to classify complications and procedures during hospitalization, including mechanical ventilation, circulatory support (e.g., intra-aortic balloon pump, left ventricular assist device, and ECMO), and the use of vasopressors.

### 2.3 Outcomes

The primary outcome of this study was the comparison of all-cause in-hospital mortality between patients with different CKM stages who were admitted to the hospital with PE. Secondary outcomes included the evaluation of in-hospital adverse events, such as major adverse cardiovascular and cerebrovascular events (MACCE), all-cause mortality, major bleeding, intracranial hemorrhage (ICH), non-ICH bleeding events, length of hospital stay, and associated costs. MACCE was defined as a composite outcome including all-cause mortality, acute ischemic stroke or transient ischemic attack, and cardiac complications. Major bleeding was categorized as a combination of gastrointestinal, retroperitoneal, intracranial, and intracerebral hemorrhages, periprocedural bleeding, unspecified bleeding, or the need for a blood transfusion. Additionally, the use of invasive management procedures—such as systemic thrombolysis, catheter-directed thrombolysis, ultrasound-facilitated catheter-directed thrombolysis, catheter-directed embolectomy, surgical embolectomy/thrombectomy, and inferior vena cava (IVC) filter placement – was also assessed.

### 2.4 Statistical analysis

Statistical analysis was conducted using IBM SPSS version 29. Continuous variables were summarized as mean, median, and interquartile range due to data skewness, while categorical variables were reported as frequencies and percentages. Pearson's chi-square test was used to compare categorical variables, and the Kruskal-Wallis test or *t*-test were applied for continuous variables, as appropriate. Sampling weights, as specified by Agency for Healthcare Research and Quality (AHRQ), were applied to estimate total discharges.

Multivariable logistic regression models were employed to assess the relationship between in-hospital outcomes and both the number and location of diseased vascular beds, with results expressed as odds ratios (OR) and 95 % confidence intervals (CI). All odds ratios were computed using CKM stage 0 as the reference group unless otherwise specified. These models were adjusted for baseline differences and controlled for covariates including age, gender, weekend admission, hospital characteristics (bed size, region, location/teaching status), and clinical factors such as high-risk PE, ventricular fibrillation (VF), ventricular tachycardia (VT), valvular heart disease, chronic liver disease, chronic lung disease, anemia, thrombocytopenia, coagulopathies, malignancies and use of various interventions (systemic thrombolysis, catheter-directed thrombolysis, ultrasound-facilitated thrombolysis, catheter-directed embolectomy, surgical embolectomy/thrombectomy, and inferior vena cava [IVC] filter placement).

## 3 Results

The study initially analyzed data from 752,960 patients with a primary diagnosis of pulmonary embolism. After applying exclusion criteria, 725,725 patients were included in the final analysis, representing 96.38 % of the original cohort. Among them, 142,670 patients (19.7 %) were classified as stage 0, 37,735 (5.2 %) as stage 1, 322,415 (44.4 %) as stage 2/3, 172,080 (23.7 %) as stage 4a, and 50,825 (7.0 %) as stage 4b. The mean age increased progressively with CKM stage, from 46.4 years in stage 1 to 72.8 years in stage 4b (p < 0.001), while the proportion of female patients decreased from 65.2 % in stage 1 to 46.3 % in stage 4b (p < 0.001). We observed a higher prevalence of high-risk PE (3.8 % in stage 1 vs. 8.2 % in stage 4b, p < 0.001) and cardiac arrest (1.3 % in stage 1 vs. 2.7 % in stage 4b, p < 0.001) at advanced stages. Comorbidities such as valvular heart disease (3.2 % in stage 1 vs. 13.3 % in stage 4b, p < 0.001), chronic lung disease (20.3 % in stage 1 vs. 35.8 % in stage 4b, p < 0.001), and anemia (22.9 % in stage 1 vs. 37.8 % in stage 4b, p < 0.001) were more prevalent at higher CKM stages. Additional baseline demographic and clinical characteristics are presented in Table 1.

**Table 1:** Baseline demographic and clinical characteristics of Pulmonary Embolism Patients stratified by CKM stage.

	CKM STAGING					P-value
	0	1	2/3	4A	4B	
NIS discharge weight	142,670	37,735	322,415	172,080	50,825	<0.001
Mean Age	52.73	46.4	64.51	68.65	72.8	<0.001
Female, %	50.8	65.2	54.2	47.3	46.3	<0.001
**Ethnicity**						<0.001
White	71.4	67.7	71.4	73.8	67.4	
Black	17.5	21.3	19.6	17.7	23.9	
Hispanic	7.0	7.4	5.7	5.2	5.1	
Asian	1.2	0.7	1.0	1.0	1.3	
Native	0.4	0.4	0.4	0.4	0.4	
Other	2.6	2.5	1.9	2.0	1.9	
**Hospital Region**						<0.001
Northeast	20.5	19.5	18.5	17.2	15.5	
Midwest or North Central	23.2	26.1	25.1	25.0	27.8	
South	36.5	36.5	39.2	40.0	38.3	
West	19.8	18.0	17.2	17.8	18.4	
**Hospital Bed Size**						<0.001
Small	21.6	20.9	20.9	20.2	20.0	
Medium	29.9	30.8	29.8	29.5	29.5	
Large	48.5	48.3	49.3	50.3	50.5	
**Hospital Location/Teaching Status**						<0.001
Rural	8.8	7.3	9.2	9.5	8.3	
Urban non-teaching	22.6	23.9	23.2	22.1	21.6	
Teaching	68.7	68.9	67.6	68.3	70.1	
**Median ZIP income**						<0.001
1st Quartile	25.7	27.7	27.8	30.8	31.1	
2nd Quartile	25.6	26.7	26.4	26.8	27.2	
3rd Quartile	25.4	25.5	25.4	24.0	24.0	
4th Quartile	23.3	20.1	20.4	18.4	17.6	
**Primary Expected Payer**						<0.001
Medicare	29.0	18.4	54.2	66.5	78.6	
Medicaid	17.4	21.5	10.4	10.5	7.2	
Private Insurance	42.7	48.9	29.3	17.9	11.3	
Self-pay	6.8	7.5	3.3	2.9	1.4	
No charge	0.6	0.6	0.3	0.2	0.1	
Other	3.6	3.1	2.5	2.1	1.6	
**Record Characteristics**						
High Risk Pulmonary Embolism	3.7	3.8	4.0	7.1	8.2	<0.001
Cardiac Arrest	1.4	1.3	1.4	2.0	2.7	<0.001
Ventricular Fibrillation	0.1	0.2	0.1	0.3	0.4	<0.001
Ventricular Tachycardia	0.5	0.6	0.8	2.6	3.2	<0.001
Cardiogenic Shock	0.9	0.9	0.9	2.4	2.6	<0.001
Saddle PE	7.2	10.5	9.8	8.5	5.4	<0.001
Acute cor pulmonale	5.8	9.3	7.6	8.9	8.3	<0.001
**Comorbidities**						
Valvular Heart Disease	3.1	3.2	4.6	10.8	13.3	<0.001
Smoking	38.7	36.4	37.9	45.4	39.9	<0.001
Dementia	2.9	0.6	5.7	8.5	10.7	<0.001
Chronic Lung Disease	17.9	20.3	23.4	34.7	35.8	<0.001
Chronic Liver Disease	0.5	0.4	0.6	0.9	1.2	<0.001
Anemia	20.8	22.9	22.2	23.5	37.8	<0.001
Thrombocytopenia	4.7	4.2	5.5	6.3	7.7	<0.001
Coagulopathy	7.7	8.9	5.9	5.9	5.0	<0.001
Homelessness	0.9	0.8	0.6	0.9	0.5	<0.001
Hematologic Malignancy	2.2	1.2	2.3	2.3	2.9	<0.001
Solid Malignancy	13.5	6.9	13.1	10.7	9.1	<0.001
Metastatic Malignancy	9.8	4.5	8.4	6.1	4.9	<0.001

Note: the mean age in stage 0 may appear higher than in stage 1 due to coding limitations and possible survivors bias.

### 3.1 In-hospital management, procedures and outcomes

**Fig. 2:**
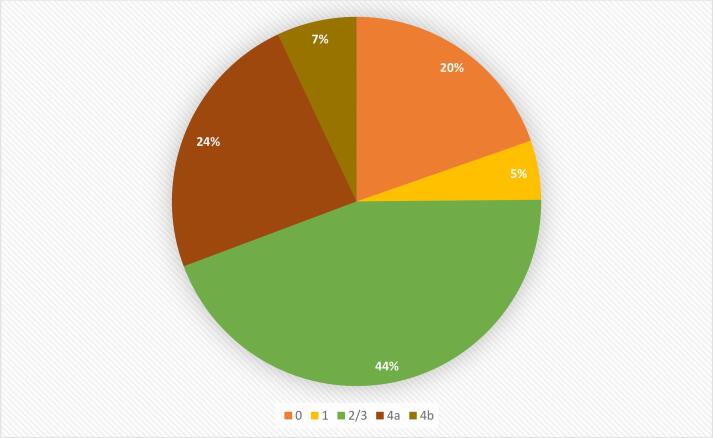
Distribution of pulmonary embolism patients according to CKM stage.

Table 2 and Fig. 2 presents significant differences in the management and outcomes patients with pulmonary embolism cross different CKM stages. In terms of management, patients with advanced CKM stages generally underwent fewer interventional procedures compared to those in earlier stages. Systemic thrombolysis decreased from 4.5 % in stage 1 to 2.6 % in stage 4B (p < 0.001). Catheter-directed thrombolysis showed a similar trend, declining from 5.1 % in stage 1 to 2.5 % in stage 4B (p < 0.001). Ultrasound-facilitated catheter-directed thrombolysis and catheter-directed embolectomy also decreased, from 1.4 % to 0.7 % and 1.2 % to 0.6 % respectively (both p < 0.001). However, IVC filter placement increased from 5.4 % in stage 1 to 7.1 % in stage 4B (p < 0.001).

**Table 2:** In-hospital procedures, complications, and outcomes of Pulmonary Embolism Patients stratified by CKM stage.

	CKM STAGING					P-value
	0	1	2/3	4A	4B	
**NIS discharge weight**	142,670	37,735	322,415	172,080	50,825	<0.001
**High Risk Pulmonary Embolism**	3.7	3.8	4.0	7.1	8.2	<0.001
**Management, %**						
Systemic thrombolysis	2.8	4.5	3.1	3.3	2.6	<0.001
Catheter-directed thrombolysis	2.8	5.1	4.1	3.4	2.5	<0.001
Ultrasound-facilitated catheter-directed thrombolysis	0.7	1.4	1.1	0.8	0.7	<0.001
Catheter-directed embolectomy	0.7	1.2	0.9	1.0	0.6	<0.001
Surgical embolectomy/ thrombectomy	0.1	0.3	0.1	0.4	0.1	<0.001
IVC filter	4.6	5.4	5.8	7.3	7.1	<0.001
**Circulatory and Ventilatory support**						
Vasopressors	0.6	0.6	0.6	1.1	1.3	<0.001
Mechanical Ventilation	2.5	2.6	2.6	4.6	5.2	<0.001
ECMO	0.1	0.1	0.1	0.3	0.1	<0.001
**Clinical outcomes, %**						
All-cause mortality	2.5	1.9	2.6	4.1	5.4	<0.001
MACCE	3.7	2.9	3.7	6.4	7.6	<0.001
Major bleeding	1.9	1.4	2.2	3.2	3.8	<0.001
ICH	0.4	0.1	0.4	0.6	0.5	<0.001
Non-ICH						
Retroperitoneal	0.2	0.1	0.2	0.2	0.3	<0.001
Gastrointestinal	1.4	1.0	1.6	2.3	2.9	<0.001
Procedure related	0.1	0.1	0.1	0.2	0.1	<0.001
Length of Stay, days, mean	3.48	4.04	4.09	5.11	5.86	<0.001
Total charge, $, mean	39954.78	47800.29	45598.64	57526.60	60073.46	<0.001

Circulatory and ventilatory support requirements increased with higher CKM stages. The use of vasopressors rose from 0.6 % in stages 0 to 1.3 % in stage 4B (p < 0.001), while mechanical ventilation rates also increased from 2.5 % in stage 0 to 5.2 % in stage 4B (p < 0.001).

Clinical outcomes worsened progressively with increasing CKM stage. All-cause mortality rose from 1.9 % in stage 1 to 5.4 % in stage 4B (p < 0.001). MACCE followed a similar pattern, increasing from 2.9 % in stage 1 to 7.6 % in stage 4B (p < 0.001), as did major bleeding events (rising from 1.4 % in stage 1 to 3.8 % in stage 4B, p < 0.001). Specifically, intracranial hemorrhage rates ranged from 0.1 % in stage 1 to 0.6 % in stage 4A (p < 0.001), while gastrointestinal bleeding increased from 1.0 % in stage 1 to 2.9 % in stage 4B (p < 0.001).

The mean length of hospital stay increased progressively with CKM stage, from 3.48 days in stage 0 to 5.86 days in stage 4B (p < 0.001). Similarly, mean total charges rose from $39,954.78 in stage 0 to $60,073.46 in stage 4B (p < 0.001).

### 3.2 Adjusted analysis

The multivariate analysis showed several significant findings (Table 3). Regarding in-hospital procedures, the odds of receiving systemic thrombolysis were highest in CKM stage 1 (aOR: 1.52, 95 % CI: 1.43–1.61, p < 0.001) and decreased progressively, with the lowest likelihood observed in stage 4B (aOR: 0.78, 95 % CI: 0.72–0.83, p < 0.001). Catheter-directed thrombolysis showed a similar pattern, with the highest likelihood in stage 1 (aOR: 1.78, 95 % CI: 1.68–1.88, p < 0.001) and lowest in stage 4B (aOR: 0.89, 95 % CI: 0.83–0.95, p = 0.001). Ultrasound-facilitated catheter-directed thrombolysis demonstrated a comparable trend, with the highest odds in stage 1 (aOR: 1.89, 95 % CI: 1.70–2.11, p < 0.001) and no significant difference in stage 4B compared to stage 0 (aOR: 0.99, 95 % CI: 0.87–1.13, p = 0.846). Catheter-directed embolectomy showed increased odds in stage 1 (aOR: 1.71, 95 % CI: 1.52–1.91, p < 0.001) and decreased odds in stage 4B (aOR: 0.65, 95 % CI: 0.57–0.75, p < 0.001). On the other hand, the likelihood of surgical embolectomy/thrombectomy was markedly elevated in stage 4A (aOR: 2.57, 95 % CI: 2.15–3.07, p < 0.001) compared to other stages. Regarding concurrent long term prophylaxis care, IVC filter use also demonstrated elevated odds in stage 1 (aOR: 1.38, 95 % CI: 1.31–1.45, p < 0.001) and showed no significant difference between stage 4B and stage 0 (aOR: 1.01, 95 % CI: 0.97–1.06, p = 0.259).

**Table 3:** Multivariate Analysis showing adjusted OR for in-hospital procedures and complications of Pulmonary Embolism Patients stratified by CKM stage.

	**CKM STAGING**							
	**1**		**2/3**		**4A**		**4B**	
**Outcome**	**aOR (95 % CI)**	**P value**	**aOR (95 % CI)**	**P value**	**aOR (95 % CI)**	**P value**	**aOR (95 % CI)**	**P value**
**In-Hospital Procedures**								
Systemic thrombolysis	1.527 (1.439–1.621)	<0.001	1.249 (1.201–1.298)	<0.001	1.148 (1.097–1.201)	<0.001	0.859 (0.802–0.919)	<0.001
Catheter-directed thrombolysis	1.788 (1.690–1.892)	<0.001	1.576 (1.518–1.636)	<0.001	1.255 (1.201–1.311)	<0.001	0.896 (0.838–0.958)	0.001
Ultrasound-facilitated catheter-directed thrombolysis	1.895 (1.700–2.112)	<0.001	1.690 (1.569–1.822)	<0.001	1.299 (1.191–1.418)	<0.001	0.993 (0.871–1.133)	0.922
Catheter-directed embolectomy	1.731 (1.546–1.939)	<0.001	1.395 (1.294–1.504)	<0.001	1.326 (1.219–1.442)	<0.001	0.673 (0.586–0.772)	<0.001
Surgical embolectomy/thrombectomy	1.977 (1.545–2.531)	<0.001	1.444 (1.203–1.733)	<0.001	2.601 (2.169–3.119)	<0.001	0.823 (0.613–1.106)	0.197
IVC filter	1.386 (1.315–1.460)	<0.001	1.104 (1.071–1.138)	<0.001	1.297 (1.255–1.341)	<0.001	1.026 (0.981–1.074)	0.259
**In-Hospital Complications**								
MACCE	0.887 (0.827–0.951)	<0.001	0.875 (0.844–0.906)	<0.001	1.369 (1.318–1.421)	<0.001	1.556 (1.483–1.633)	<0.001
Mortality	0.867 (0.795–0.947)	0.001	0.805 (0.772–0.841)	<0.001	1.069 (1.021–1.119)	0.004	1.365 (1.288–1.446)	<0.001
Major Bleeding	0.742 (0.674–0.818)	<0.001	0.979 (0.934–1.026)	0.376	1.219 (1.159–1.283)	<0.001	1.153 (1.080–1.231)	<0.001
ICH	0.422 (0.319–0.559)	<0.001	1.033 (0.932–1.145)	0.533	1.200 (1.072–1.343)	0.002	1.076 (0.918–1.262)	0.364

Reference: CKM STAGE 0; adjusted for age, gender, weekend admission, hospital bed size, region and location/teaching status, cardiogenic shock, VF, VT, AF, HF, hypertension, valvular heart disease, dyslipidemia, chronic liver disease, chronic lung disease, chronic kidney disease, anemia, thrombocytopenia, coagulopathies, diabetes mellitus, malignancies, systemic thrombolysis, catheter-directed therapy, surgical embolectomy/thrombectomy and IVC filter.

In terms of clinical outcomes, a paradoxical trend was observed. The odds of mortality decreased with advancing CKM stages, with the lowest risk in stage 4A (aOR: 0.28, 95 % CI: 0.27–0.29). MACCE showed consistently lower odds across all CKM stages compared to the Stage 0, with the lowest in stage 4A (OR: 0.48, 95 % CI: 0.47–0.49). Acute CVA did not demonstrate a clear linear relationship with CKM severity, with the lowest odds in stage 1 (OR: 0.53, 95 % CI: 0.48–0.58). Major bleeding events also showed lower odds across all CKM stages, with the lowest in stage 4B (OR: 0.65, 95 % CI: 0.62–0.68). Notably, all reported associations were statistically significant (p-value < 0.001). All odds ratios are referenced to CKM stage 0 patients.

### 3.3 Disposition of patients

**Fig. 3:**
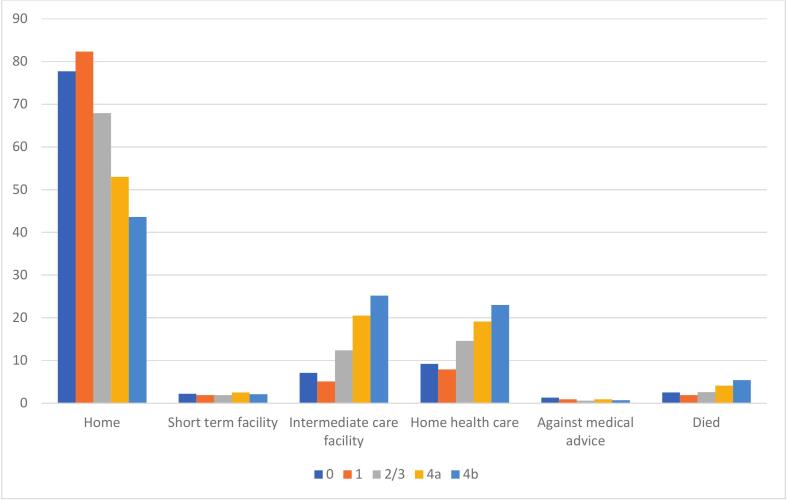
Disposition of pulmonary embolism patients according to CKM stage.

Fig. 3 represents the disposition of patients with AMI across different stages of CKM. The majority of patients across all stages were discharged home, with the highest percentage observed in Stage 1 (63.9 %) and the lowest in Stage 4B (39.1 %). Interestingly, the need for intermediate care facilities and home health care increased with disease progression, peaking at 25.2 % and 18 % respectively for Stage 4B patients. Short-term facility utilization was highest in Stage 1 (13.2 %) and lowest in Stage 4B (6.6 %). And the percentage of patients leaving against medical advice was relatively low across all stages, with a maximum of 2.9 % in Stage 0.

## 4 Discussion

Our analysis of 725,725 PE patients revealed several key findings regarding the impact of CKM syndrome staging on clinical outcomes and management. First, patients with advanced CKM were more likely to be male, older, and to present with ventricular arrhythmias, and high-risk PE. Second, resource utilization, as measured by length of hospital stay and total charges, showed a consistent upward trend with advancing CKM stages. Third, after adjustments, our multivariate analysis revealed significant associations between CKM staging and both management approaches and clinical outcomes. Patients in higher CKM stages had lower odds of receiving some invasive treatments such as systemic thrombolysis and catheter-directed interventions, but were more likely to undergo surgical embolectomy/thrombectomy and to receive IVC filters. Furthermore, the risk of adverse clinical outcomes increased with higher CKM stages, with patients in advanced stages facing significantly higher odds of MACCE, all-cause in-hospital mortality and major bleeding.

The observed demographic shift towards older, predominantly male patients in advanced CKM stages is consistent with previous studies on PE. Multiple studies have demonstrated that the risk of venous thromboembolism (VTE) increases markedly with age, showing an exponential rise in incidence among older populations [[14], [15], [16]]. For instance, according to Gregson et al., the risk of VTE increased approximately 2.8-fold per decade in the Emerging Risk Factors Collaboration (ERFC) data and 1.8-fold per decade in the UK Biobank data [14]. Additionally, at older ages, men are at a modestly greater risk of VTE than women [[17], [18], [19]]. Heit et al., found that while the overall age-adjusted incidence rate of VTE is higher for men (130 per 100,000) than women (110 per 100,000), with a male:female sex ratio of 1.2:1, this difference becomes more pronounced with advancing age [17]. The increased risk of VTE, including PE, in older populations is likely multifactorial. There is a gradual increase in procoagulant factors, such as fibrinogen, factor VII, factor VIII, and factor IX, coupled with a decrease in anticoagulant proteins like antithrombin shifting towards a more prothrombotic state increases the risk of clot formation [20]. Furthermore, older individuals often have reduced mobility, which can lead to venous stasis and increased risk of thrombosis [21]. In our statistical analysis we found out that there is a slightly higher mean age in stage 0 compared to stage 1. This likely reflects a survivor bias and limitations in administrative data coding, as discussed later in detail in the limitation section.

There is currently limited research directly linking CKM syndrome and PE. The risk factors associated with CKM syndrome, particularly obesity, diabetes, and chronic kidney disease (CKD), are strongly linked to an increased incidence of pulmonary embolism [[4], [5], [6]]. These conditions create a pro-thrombotic environment that promotes clot formation through various mechanisms, including inflammation and endothelial dysfunction [22]. The interplay between these risk factors often leads to a compounding effect, as they frequently coexist with additional comorbidities, further exacerbating patient outcomes.

Obesity is a significant contributor to the development of PE and other adverse cardiovascular outcomes. A study by Goldhaber et al. [4] investigated risk factors for PE in women and found that those with a body mass index (BMI) of 29 kg/m^2^ or higher had an age-adjusted relative risk (RR) of 3.2 for primary PE compared to women in the leanest category (BMI < 21 kg/m^2^). Furthermore, Beenan et al. [23] demonstrated a U-shaped relationship between BMI categories and both prognostic parameters and clinical outcomes in patients with PE. These findings suggest that extreme obesity may exacerbate the hemodynamic consequences of PE, potentially leading to poorer outcomes.

CKD, another key component of CKM syndrome, demonstrate a significantly higher incidence of PE and worse outcomes compared to those with normal kidney function. Singh et al. found that patients with PE with CKD had a mortality rate of 4.5 %, while those with ESRD had a mortality rate of 6.8 %, compared to 2.7 % in patients with normal kidney function [24]. The heightened thrombotic risk in patients with CKD is attributed to various factors, including increased levels of procoagulant markers, decreased endogenous anticoagulants, enhanced platelet activation and aggregation, and reduced fibrinolytic system activity [25].

Diabetes mellitus, another element of CKM syndrome, significantly influences PE risk and outcomes. A comprehensive analysis of 1,174,196 PE patients by Schmitt et al. revealed that 18.7 % had diabetes, and these patients exhibited a higher in-hospital mortality rate compared to patients with no diabetes (19.8 % vs 14.8 %, P < 0.001). Importantly, diabetes was independently associated with increased in-hospital mortality (odds ratio [OR] 1.21, 95 % confidence interval [CI] 1.20–1.23, P < 0.001) when adjusted for age, sex, and comorbidities [26]. The pathophysiological mechanisms underlying the increased risk of PE in patients with diabetes are multifaceted which includes chronic hyperglycemia, systemic inflammation, and heightened platelet aggregation [27]. These risks are further exacerbated by comorbid conditions frequently seen in patients with diabetes, including obesity, dyslipidemia, and reduced mobility due to neuropathy. Together, these factors create a synergistic effect that predisposes individuals to DVT and PE.

The observed trend of decreased use of invasive treatments in higher CKM stages aligns with findings from studies on PE management in patients with multiple comorbidities. In a comprehensive analysis by Truong-An Ho et al., patients with higher Charlson Comorbidity Index (CCI) scores, indicating multiple comorbidities, were indeed less likely to receive advanced therapies for PE. Specifically, patients with CCI ≥ 3 were less likely to receive systemic thrombolysis, catheter-directed thrombolysis, and mechanical thrombectomy compared to those with lower CCI scores [28]. This trend is consistent with the concept that patients with higher comorbidity burdens may be at increased risk for complications from invasive procedures, leading clinicians to opt for more conservative management strategies in these complex cases.

The increase in adverse clinical outcomes with advancing CKM stages in our study is corroborated by several investigations examining the impact of individual CKM components on PE outcomes. Schmitt et al. [26] demonstrated that diabetes was independently associated with increased in-hospital mortality in patients with PE (OR 1.21, 95 % CI 1.20–1.23, p < 0.001). Similarly, a study by Singh et al. [24] found that patients with PE, with CKD and ESRD had significantly higher mortality rates (4.5 % and 6.8 %, respectively) compared to those with normal kidney function (2.7 %). The complex relationship between individual CKM components and PE outcomes underscores the importance of considering the cumulative effect of multiple comorbidities, as captured by CKM staging, in predicting and managing PE outcomes. As evaluation and management of cardiovascular risks shifts to incorporating multimorbidity conditions with shared risk factors such as CKM staging, the outcome of the current study helps provide important insights into the role of CKM syndrome on management and outcomes of the PE population.

Our study provides meaningful insights into how different CKM stages influence outcomes in patients with PE, but it is important to address certain limitations. First, as a retrospective analysis based on administrative data, it is prone to coding errors and lacks detailed clinical context that could enrich the findings. Second, the study does not differentiate between the individual causes and comorbidities within each CKM stage, which may have varying effects on PE outcomes. Third, it does not account for confounding variables like medication use, which could influence both CKM-related symptoms and thrombotic risks. Fourth, there is a significant potential for undercoding of CKM stages, particularly for stages 1–4, which may have led to an overestimation of stage 0 prevalence in our study population. Furthermore, Stage 0 comprises patients without documented CKM risk factors, potentially representing an older, healthier subgroup or reflecting incomplete coding of early risk factors in younger patients. This is noted to caution interpretation and is consistent with similar findings in large electronic health record datasets. Fifth, the specific ICD-10 codes used to define each CKM stage may not fully capture the complexity and nuances of the syndrome.

Despite these limitations, the study has significant strengths, including a large sample size that provides strong statistical power and the use of a nationally representative database, which enhances the generalizability of its findings. Furthermore, its comprehensive evaluation of multiple aspects of PE management and outcomes offers a nuanced understanding of the impact of different CKM stages on PE prognosis.

Although advanced CKM stages encompass many conventional risk factors, including older age and common comorbidities, the CKM classification provides a unified assessment of interconnected organ dysfunction. Emerging evidence suggests that broader multimorbidity indices, such as CKM, can identify high-risk subgroups not otherwise apparent from age or single-organ disease measures alone [8,9,28].

## 5 Conclusion

Our comprehensive analysis of patients with PE revealed the significant impact of CKM syndrome staging on clinical outcomes and management strategies. As CKM stages advanced, patients faced progressively higher risks of adverse events, including increased mortality and major bleeding complications. Paradoxically, these high-risk patients were less likely to receive invasive treatments, highlighting a critical gap in care. Our findings underscore the urgent need for tailored management approaches that consider the complex interplay of comorbidities in CKM syndrome. By implementing targeted interventions and risk stratification strategies, healthcare providers can potentially improve outcomes and reduce the substantial burden of PE in this vulnerable population.

## Funding statement

Not applicable.

## CRediT authorship contribution statement

**Shay Zvi Cherevatsky:** Writing – original draft. **Marlon V. Gatuz:** Writing – original draft. **Adam Folman:** Writing – review & editing. **Maguli S. Barel:** Writing – review & editing. **Rami Abu-Fanne:** Writing – review & editing. **Dmitry Abramov:** Writing – review & editing. **Mamas A. Mamas:** Writing – review & editing. **Ariel Roguin:** Writing – review & editing. **Ofer Kobo:** Writing – original draft.

## Declaration of competing interest

The authors declare that they have no known competing financial interests or personal relationships that could have appeared to influence the work reported in this paper. In addition, there was no funding to this research.
