# Supplementary material for: Impact of cardiovascular-kidney-metabolic syndrome staging on clinical outcomes and management of acute pulmonary embolism: A comprehensive analysis
Source: Int J Cardiol Heart Vasc. 2025 Oct 30;61:101831. doi: 10.1016/j.ijcha.2025.101831 (PMC12607129; doi:10.1016/j.ijcha.2025.101831)
Supplement: Supplementary Data 1 [file mmc1.docx]

**Table S1: ICD-10 codes for patient characteristics, in hospital procedures and post-procedural complications**

| **Variable** | **Codes** |
| --- | --- |
| **Patient and Record Characteristics** | |
| Dementia | D631, E0822, E0922, E1022, E1122, E1322, I120, I129, I130, I1310, I1311, I132, N181, N182, N183, N184, N185, N186, N189, R880, Z4901, Z4902, Z4931, Z4932 |
| Cardiac Arrest | I46.x |
| Ventricular Fibrillation | I49.01 |
| Ventricular tachycardia | I47.2 |
| Cardiogenic Shock | R57.0 |
| Valvular disease | I05-08; I34-37 |
| Smoking | Z87.891 , Z72.0 |
| Anemia | D55-59 |
| Thrombocytopenia | D69.3-.6 |
| Coagulopathy | D65-68;D69.0-.2 |
| Chronic Liver Disease | K73-74 |
| Homelessness | Z59.0 |
| Saddle PE | I2602, I2692, I2609,I260, I2699 |
| Acute cor pulmonale | I2601, I2602, I2609 |

| **In Hospital Procedures / Outcomes** | |
| --- | --- |
| Systemic thrombolysis | 3E03317 |
| Catheter-directed thrombolysis | 3E06317 |
| Ultrasound-facilitated catheter-directed thrombolysis | 6A750Z5 ,6A750Z6, 6A750Z7, 6A750ZZ, 6A751Z5, 6A751Z6, 6A751Z7, 6A751ZZ |
| Catheter-directed embolectomy | 02CP3ZZ, 02CQ3ZZ, 02CR3ZZ |
| Surgical embolectomy | 02CP0ZZ, 02CQ0ZZ, 02CR0ZZ |
| Mechanical Ventilation | 5A19054/35Z/45Z/55Z |
| Use of vasopressors | 3E030XZ, 3E033XZ, 3E040XZ, 3E043XZ |
| Circulatory support (inc. IABP, LV assist device and ECMO). | 5A02x, 5A1522G, 5A15A2G, 5A15A2H |
| GI bleed | K92.0-92.2; K25.0-25.2; K25.4-25.6; K26.0-  26.2; K27.0-27.2; K27.4-27.6; K28.0-28.2; K28.4-28.6 |
| Retroperitoneal Bleed | K66.1 |
| Intracranial Hemorrhage | I60-62 |

**Table S2: Staging system of Cardiovascular-Kidney-Metabolic (CKM) syndrome, categorized by American Heart Association (AHA)**

| **CKM syndrome stages** | **Definition** |
| --- | --- |
| Stage 0: No CKM risk factors | Individuals with normal BMI and waist circumference, normoglycemia, normotension, a normal lipid profile, and no evidence of CKD or subclinical or clinical CVD |
| Stage 1: Excess or dysfunctional adiposity | Individuals with overweight/obesity, abdominal obesity, or dysfunctional adipose tissue, without the presence of other metabolic risk factors or CKD  BMI ≥25 kg/m^2^ (or ≥23 kg/m^2^ if Asian ancestry),  Waist circumference ≥88/102 cm in women/men (or if Asian ancestry ≥80/90 cm in women/men), or  Fasting blood glucose ≥100–124 mg/dL or HbA1c between 5.7% and 6.4%* |
| Stage 2: Metabolic risk factors and CKD | Individuals with metabolic risk factors (hypertriglyceridemia [≥135 mg/dL], hypertension, MetS†, diabetes), or CKD |
| Stage 3: Subclinical CVD in CKM | Subclinical ASCVD or subclinical HF among individuals with excess/dysfunctional adiposity, other metabolic risk factors, or CKD  Subclinical ASCVD to be principally diagnosed by coronary artery calcification (subclinical atherosclerosis by coronary catheterization/CT angiography also meets criteria)  Subclinical HF diagnosed by elevated cardiac biomarkers (NT-proBNP ≥125 pg/mL, hs-troponin T ≥14 ng/L for women and ≥22 ng/L for men, hs-troponin I ≥10 ng/L for women and ≥12 ng/L for men) or by echocardiographic parameters, with a combination of the 2 indicating highest HF risk. Risk equivalents of subclinical CVD  Very high-risk CKD (stage G4 or G5 CKD or very high risk per KDIGO classification)  High predicted 10-y CVD risk |
| Stage 4: Clinical CVD in CKM | Clinical CVD (coronary heart disease, HF, stroke, peripheral artery disease, atrial fibrillation) among individuals with excess/dysfunctional adiposity, other CKM risk factors, or CKD  Stage 4a: no kidney failure  Stage 4b: kidney failure present |

ASCVD indicate atherosclerotic cardiovascular disease; BMI, body mass index; CKD, chronic kidney disease; CKM, cardiovascular-kidney-metabolic; CT, computed tomography; CVD; cardiovascular disease; HbA1c, hemoglobin A1c; HDL, high-density lipoprotein; HF, heart failure; hs-troponin, high sensitivity troponin; KDIGO; Kidney Disease Improving Global Outcomes; MetS, metabolic syndrome; and Nt-proBNP, N-terminal pro-B-type natriuretic peptide.

* Individuals with gestational diabetes should receive intensified screening for impaired glucose tolerance after pregnancy.

† MetS is defined by the presence of 3 or more of the following: (1) waist circumference $\geq$ 88 cm for women and $\geq$ 102 cm foe men ($\geq$ 80 cm for women or $\geq$ 90 cm for men if Asian ancestry); (2) HDL cholesterol < 40 mg/dL for men and < 50 mg/dL for women; (3) triglycerides $\geq$ 150 mg/dL; (4) elevated blood pressure (systolic blood pressure $\geq$ 130 mm Hg or diastolic blood pressure $\geq$ 80 mm Hg and/or use of antihypertensive medications); and (5) fasting blood glucose $\geq$ 100 mg/dL.
